# Garlic-YOLO-DD: a lightweight object detection algorithm for garlic damage detection

**DOI:** 10.3389/fpls.2025.1702045

**Published:** 2026-01-06

**Authors:** Yun Gao, Xiaodan Ma, Zhennan Xia, Tao Qi, Xin Wang, Zhuang He, Gang Chen

**Affiliations:** School of Information Engineering, Changchun College of Electronic Technology, Changchun, China

**Keywords:** garlic damage detection, lightweight network, object detection, precision agriculture, YOLO

## Abstract

To address the challenge of applying garlic damage detection models in resource-constrained environments, this study proposes Garlic-YOLO-DD—a lightweight single-stage object detection algorithm based on YOLOv11n. This model effectively resolves the core issues of high computational complexity and excessive parameters in existing methods, achieving efficient and accurate garlic damage recognition suitable for real-time applications. Specifically, replacing conventional convolutional modules in the backbone network with the ADown module significantly reduces parameters and computational load. Simultaneously, integrating the parameter-free SimAM attention mechanism enhances localization and feature extraction capabilities for subtle lesion areas. The efficient BiFPN architecture optimizes the original feature fusion network, improving both speed and effectiveness in multi-scale feature integration. Experiments conducted on a self-built garlic damage dataset demonstrate that the Garlic-YOLO-DD model reduces the number of parameters to 57.96% of YOLOv11n, decreases computational load by 20.63%, increases inference speed by 15.97%, and achieves mAP@50% by 27.64%. This study provides a computer vision solution for automated garlic damage detection in intelligent agricultural systems.

## 1 Introduction

Garlic, as a globally cultivated and important cash crop, has its quality directly impacting agricultural profitability and market value (Puspitasari et al., 2024; Martínez-López et al., 2022; Anum et al., 2024; Song et al., 2025). During post-harvest handling, garlic bulbs are highly susceptible to damage from mechanical operations or environmental factors (Madhu et al., 2025; 2019a; Kale et al., 2024; Tullo, 2023; Makarichian et al., 2021; Li et al., 2024; Makarichian et al., 2025). This damage not only diminishes garlic’s commercial value but also accelerates decay and spoilage, posing significant food safety concerns (Tavoni et al., 2024). Hence, achieving rapid and precise detection of garlic damage is crucial for safeguarding agricultural product quality and advancing automated processing (Wu et al., 2019; Jia et al., 2020; Raki et al., 2024).

In recent years, deep learning-based object detection techniques have demonstrated significant potential in the field of agricultural visual inspection (Dalal and Mittal, 2025; Kanna et al., 2024; Gong and Wu, 2025; Silva et al., 2024; Pagire et al., 2025; Badgujar et al., 2024; Liu et al., 2025; Zhao et al., 2021). However, existing high-performance models typically involve high computational complexity and massive parameter scales, while also relying on expensive GPU computing power (Cao et al., 2023; Chen et al., 2025). This severely limits the practical application of these models in resource-constrained scenarios, such as embedded devices, mobile terminals, or on-site real-time detection environments (Lazarou and Exarchos, 2024; Liu et al., 2025; Yang et al., 2024). Therefore, developing a lightweight damage detection model that balances high accuracy with low resource consumption has become a critical challenge for advancing the practical implementation of intelligent agricultural detection technologies (Yang et al., 2024; Islam et al., 2025; Rezk et al., 2025; Choe and Lee, 2023).

Prior studies have explored YOLO models for mold detection (Sun et al., 2022) and crop detection in field settings (Lan et al., 2024). Some scholars applied hyperspectral imaging (Ge et al., 2024; Jin et al., 2022) or transformer-based architectures (Bi et al., 2022) for crop classification. Some scholars have also attempted to identify and classify the quality information of crop by improving the traditional convolutional neural network or proposing a brand-new image classification network (Yu et al., 2025; Qi et al., 2023; Zhao et al., 2023; Yu et al., 2025; Koklu et al., 2021; Yu et al., 2024; Joshi et al., 2021; Song et al., 2025; Zhang et al., 2025; Sun et al., 2024; Wang et al., 2024). While these methods achieve high accuracy, they often rely on computationally intensive models or costly imaging systems, which hinder their practical application in real-world production settings.

The primary contributions of this work are delineated as follows:

1. We present Garlic-YOLO-DD, an algorithm that innovates on the YOLOv11n (Khanam and Hussain, 2024) architecture to address the critical need for low-latency, high-precision garlic damage detection in practical agricultural settings.
2. To alleviate computational burdens, we architect a more efficient backbone by substituting standard convolutions with the lightweight ADown subsampling module, leading to a substantial reduction in model size and operations.
3. We incorporate the SimAM attention module, which operates without introducing additional parameters, to augment the network’s capacity for capturing discriminative features of small and inconspicuous damages, thereby elevating localization precision.
4. The original feature fusion network is refined by adopting the BiFPN paradigm, which facilitates superior cross-scale connections and contributes notably to the acceleration of inference and the enhancement of detection performance.

## 2 Materials and methods

### 2.1 Data collection and preprocessing

**Figure 1:**
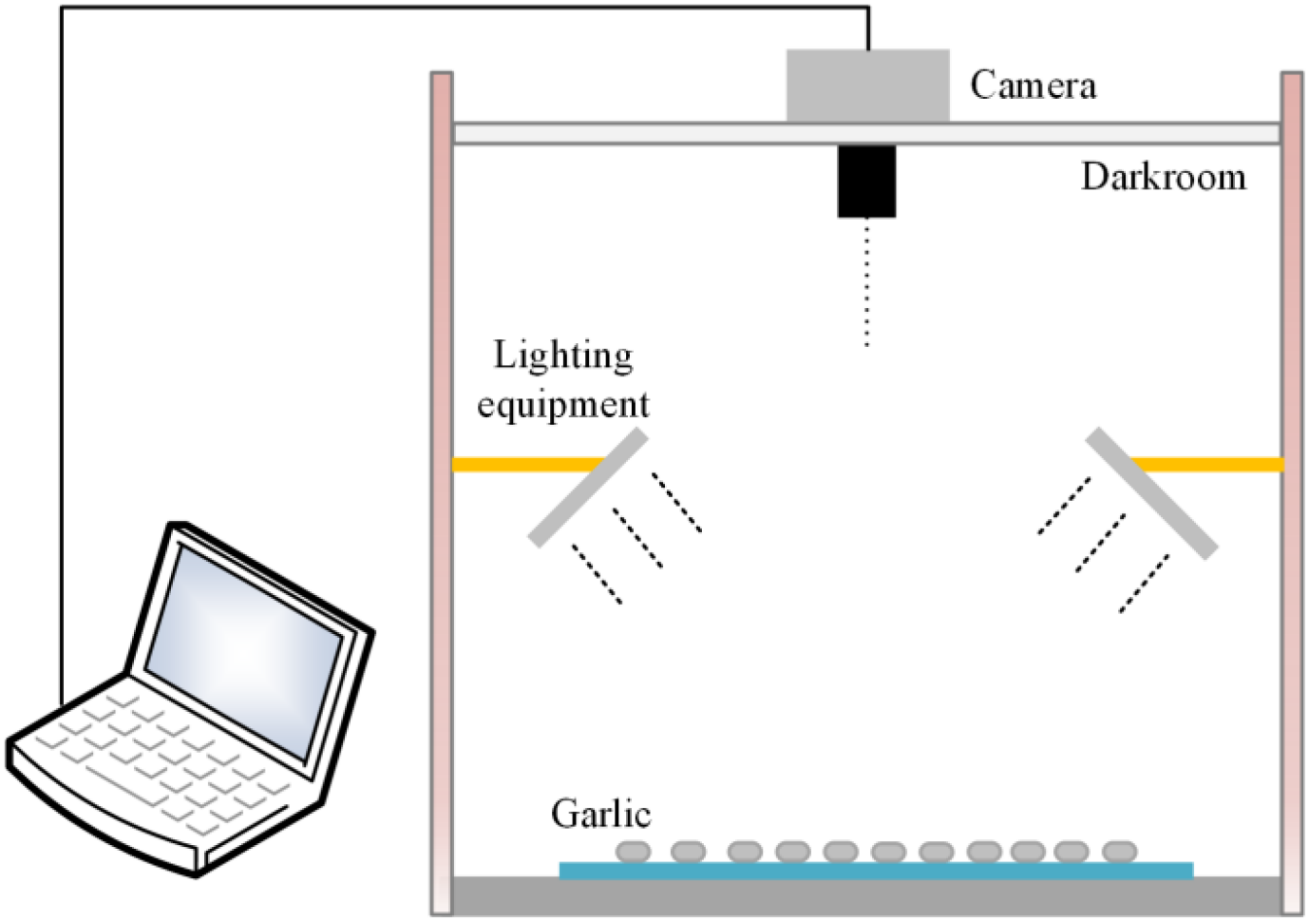
Garlic image capture system.

During image acquisition, this study employed the Honor Magic6 smartphone as the imaging device, with the experimental setup illustrated in **Figure 1**. Experiments were conducted in a darkroom to eliminate ambient light interference, utilizing only two sets of bidirectional controllable light sources for illumination to ensure stable and uniform lighting conditions. This configuration effectively minimized shadows and reflections caused by ambient light, guaranteeing the acquisition of high-quality images. Garlic samples (Variety Fengchan No. 1, sourced from Heze City, Shandong Province, China) were randomly placed on a black light-absorbing cloth to simulate their arrangement on a conveyor belt. The image acquisition device was mounted vertically on a top bracket to ensure consistency in image capture. All garlic datasets constructed in this study were annotated with bounding boxes using the online tool Make Sense.

**Figure 2:**
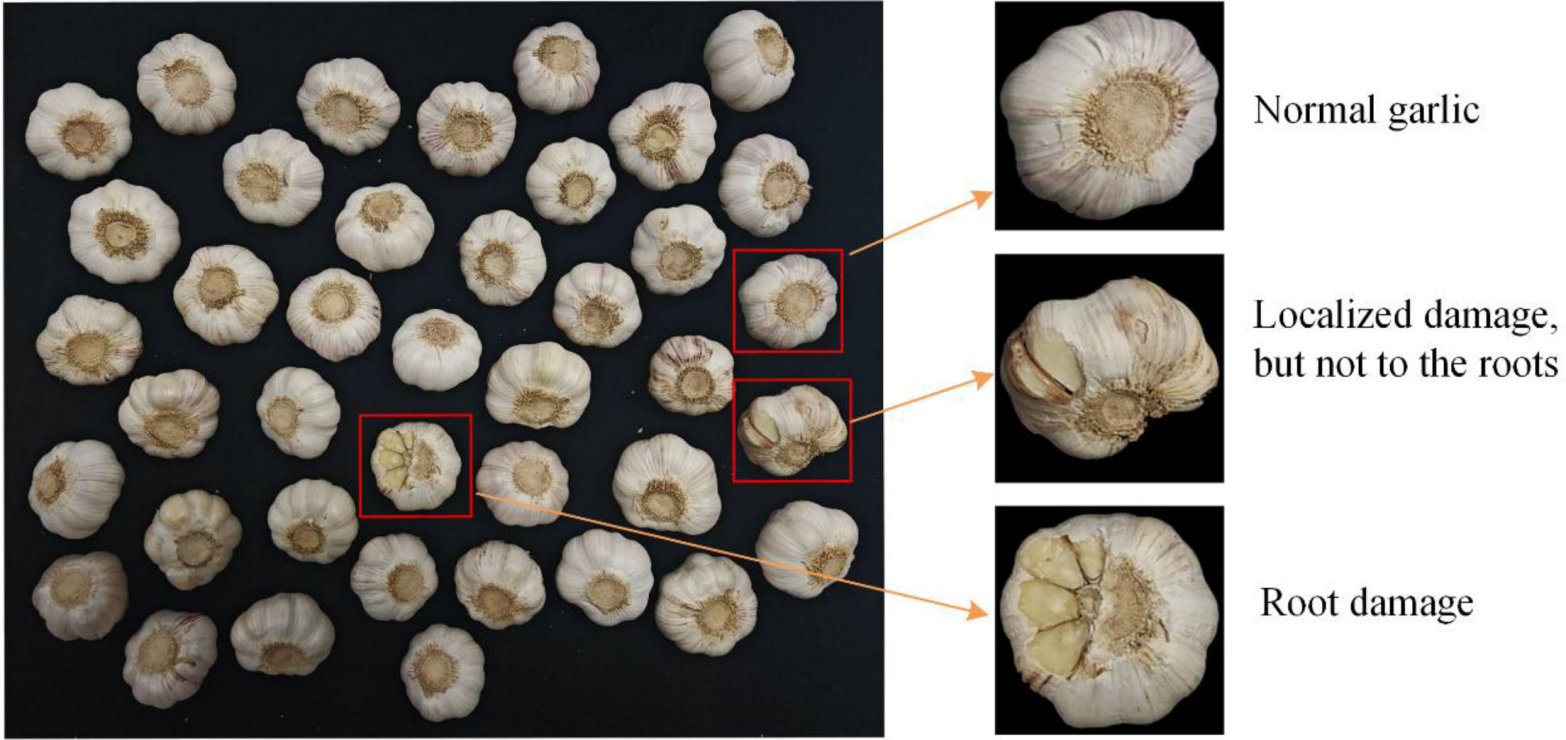
Garlic image data display.

**Figure 2** clearly demonstrates the effectiveness of the garlic dataset constructed in this study. This dataset comprises 462 high-resolution images, randomly partitioned into training, validation, and testing sets at a ratio of 7:2:1. All images were uniformly resized to 640×640 pixels before inputting into the model. To enhance model generalization, random rotation augmentation—a built-in technique in the YOLO model—was applied during training. Each garlic instance was annotated into one of three categories. A black light-absorbing cloth was used as the background to simulate the industrial scenario of conveyor belt transportation. This design reduces visual noise, enabling the model to focus more effectively on damage features and thereby enhancing the practical application value of the dataset.

Normal garlic samples: These garlic bulbs exhibit an intact appearance with compact, plump cloves and undamaged skin. Such garlic qualifies as high-quality agricultural produce with significant market value (Madhu et al., 2019b).

Partially Damaged Garlic (intact root system): These bulbs exhibit superficial blemishes such as minor skin abrasions, surface scratches, or slight clove deformation (Wang et al., 2025). Damage is confined to the exterior, with the internal structure and root system remaining intact. Despite visible cosmetic flaws, structural integrity and functionality are largely preserved, retaining some edible and commercial value.

Garlic with root damage: These samples exhibit significant root damage, including breakage or rot (Yang et al., 2024). Damaged roots impede water and nutrient absorption, ultimately leading to quality deterioration and reduced shelf life. Additionally, bulbs with root damage are unsuitable for planting and possess lower commercial value.

### 2.2 Model construction

#### 2.2.1 YOLOv11n

**Figure 3:**
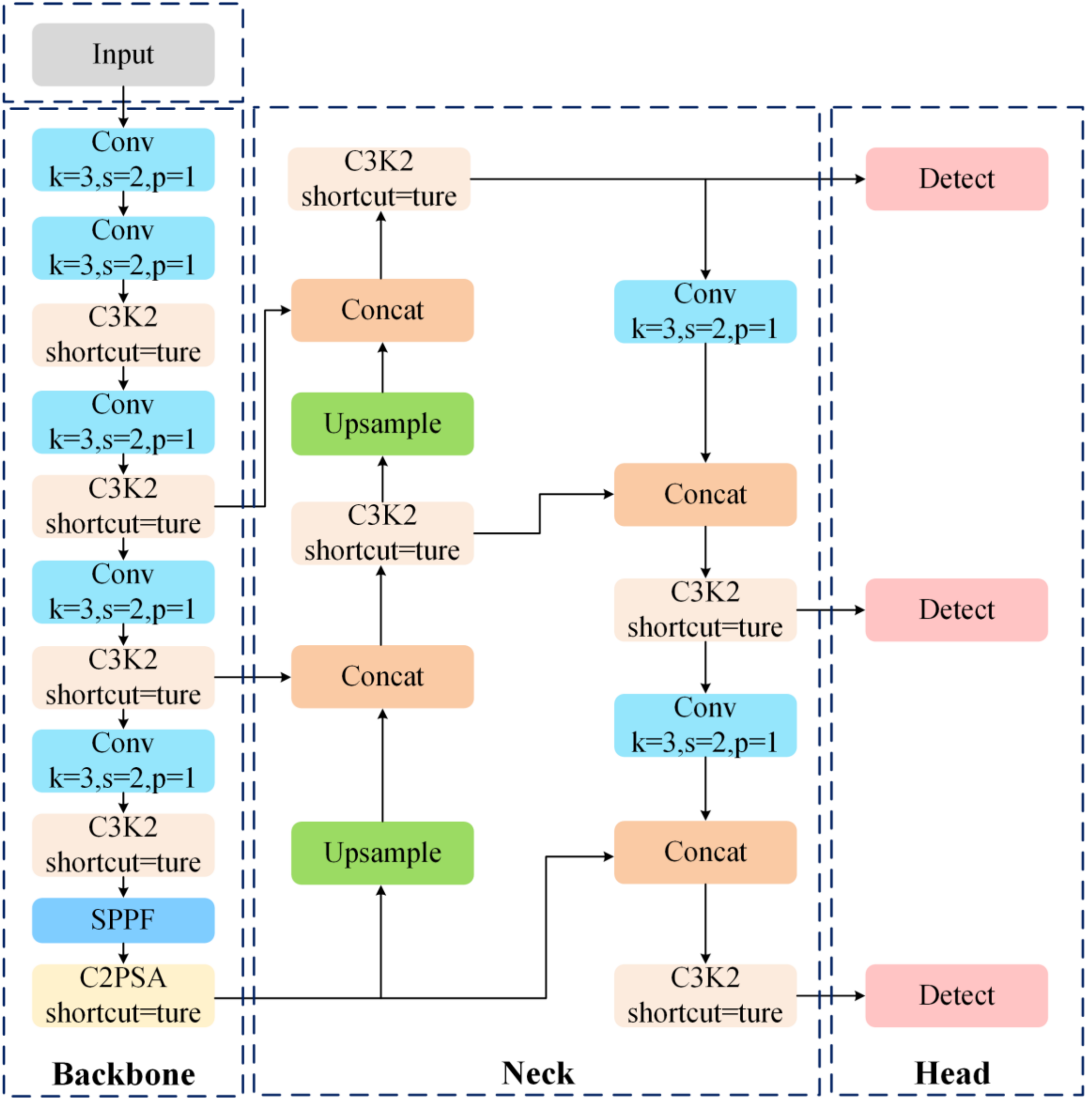
The architecture of YOLOv11n.

**Figure 3** clearly illustrates the overall architecture of the base model YOLOv11n adopted in this study. As a lightweight variant within the YOLOv11 series, this model still adheres to the mature “backbone-neck-head” framework. To maintain low computational overhead, the network employs depthwise separable convolutions and cross-stage local modules, while simultaneously promoting efficient gradient flow during backpropagation. The backbone network employs multi-scale feature propagation, while the neck module utilizes an enhanced Feature Pyramid Network (FPN). This module achieves feature fusion through bidirectional top-down and bottom-up paths, effectively integrating fine-grained information from lower layers with rich semantic information from higher layers. This significantly enhances the model’s ability to recognize garlic damage across scales. In the head module, YOLOv11n employs a decoupled design, separating the classification task and bounding box regression task into independent branches. This design reduces task interference caused by feature sharing, thereby improving detection accuracy. The final prediction layer outputs confidence scores, class probabilities, and bounding box coordinates for each detected object.

#### 2.2.2 Garlic-YOLO-DD

**Figure 4:**
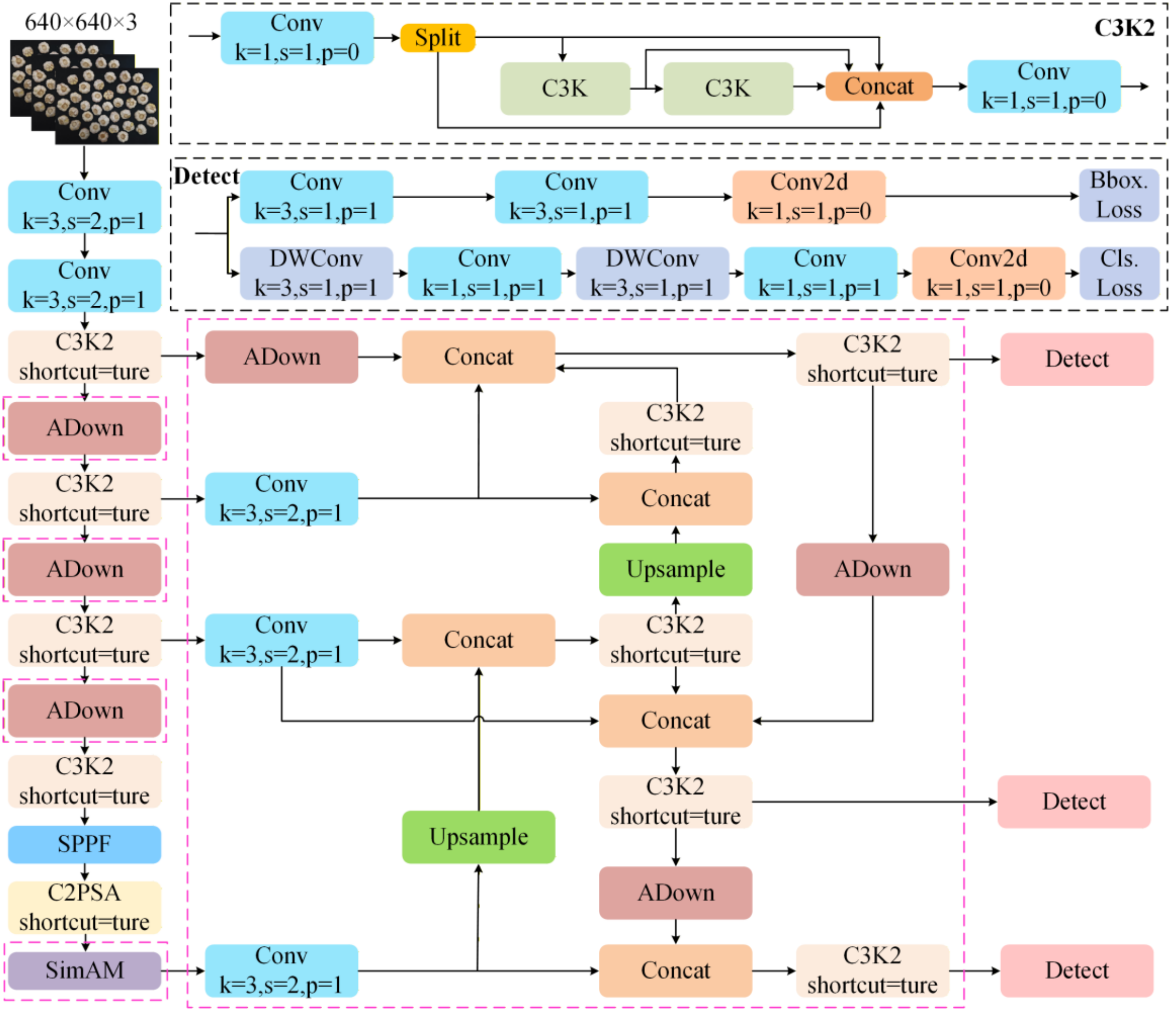
The architecture of Garlic-YOLO-DD.

In this study, we implemented three enhancement strategies on the baseline model YOLOv11n to improve its performance in garlic disease detection, as shown in **Figure 4**. First, we introduced the lightweight ADown module to replace conventional convolutional modules in the backbone network. This approach significantly reduces computational cost and parameter size while enhancing multi-scale representation capabilities. Second, a parameter-free SimAM attention module was introduced at the end of the backbone network. Driven by an energy function mechanism, this module enhances the network’s ability to focus on damaged areas and improves localization accuracy for subtle defect features. Finally, in the feature fusion stage, the original path aggregation network was replaced with a weighted bidirectional feature pyramid network (BiFPN). This design achieves more adaptive multi-scale fusion through bidirectional cross-scale connections and learnable feature weights, strengthening detection performance across different damage scales. These synergistic optimizations enable the model to maintain a lightweight design while achieving higher accuracy and more efficient inference capabilities.

#### 2.2.3 ADown module

**Figure 5:**
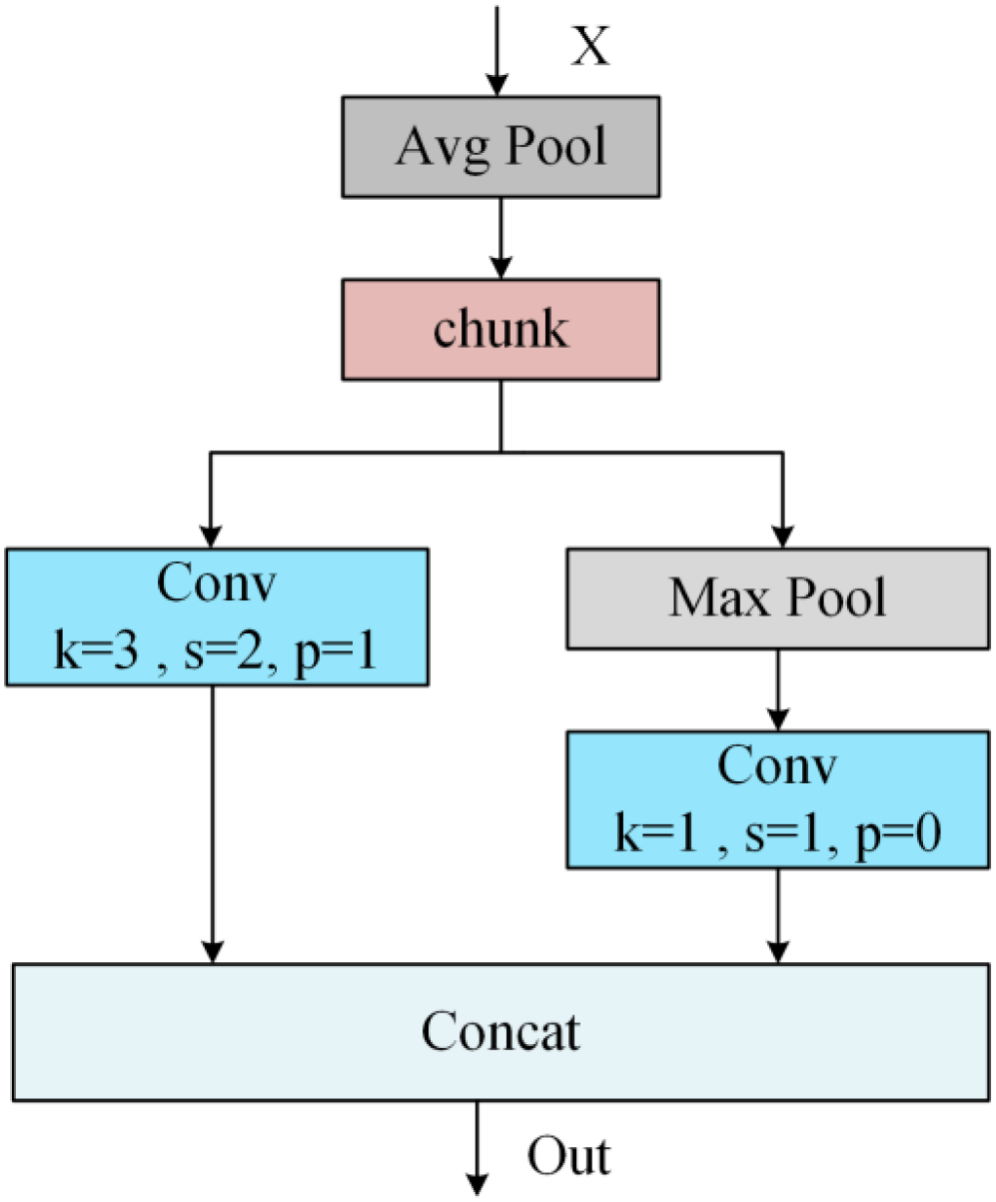
The architecture of ADown.

The ADown module (Asymmetric Downsampling) (Wang et al., 2024) is an efficient asymmetric downsampling architecture first introduced in YOLOv9 to mitigate the information loss commonly associated with traditional downsampling during feature map compression. ADown achieves more efficient downsampling while preserving richer feature information by decoupling spatial reduction from channel expansion. As shown in **Figure 5**, its core employs a parallel branch design: one branch performs spatial downsampling via asymmetric convolutions, while the other branch preserves feature responses through pooling operations. The outputs from both branches are subsequently fused. This asymmetric decomposition significantly reduces computational parameters while diversifying the receptive field. Subsequent experimental results confirm this conclusion: ADown maintains subsampling efficiency while more effectively preserving fine-grained features. It is particularly well-suited for visual tasks requiring precise detection of minute objects, such as identifying damage in garlic.

#### 2.2.4 BiFPN architecture

BiFPN (Weighted Bi-directional Feature Pyramid Network) (Tan et al., 2020) is a weighted bidirectional feature pyramid network whose core concept is to optimize multi-scale feature fusion through efficient cross-scale connections and learnable feature weights. This architecture introduces three key improvements over traditional FPN (top-down) and PANet (bottom-up) structures: First, it removes nodes with only one input edge, simplifying the network structure. Second, it adds skip connections between input and output nodes at the same scale to promote feature reuse. Finally, it introduces learnable weight parameters to perform adaptive weighted fusion based on the importance of different input features. This design enables BiFPN to efficiently aggregate feature maps at different resolutions with low computational cost, enhancing the network’s representation capability for multi-scale objects. Experiments demonstrate that BiFPN significantly reduces computational overhead while maintaining high accuracy, making it highly suitable for integration into lightweight object detection models.

**Figure 6:**
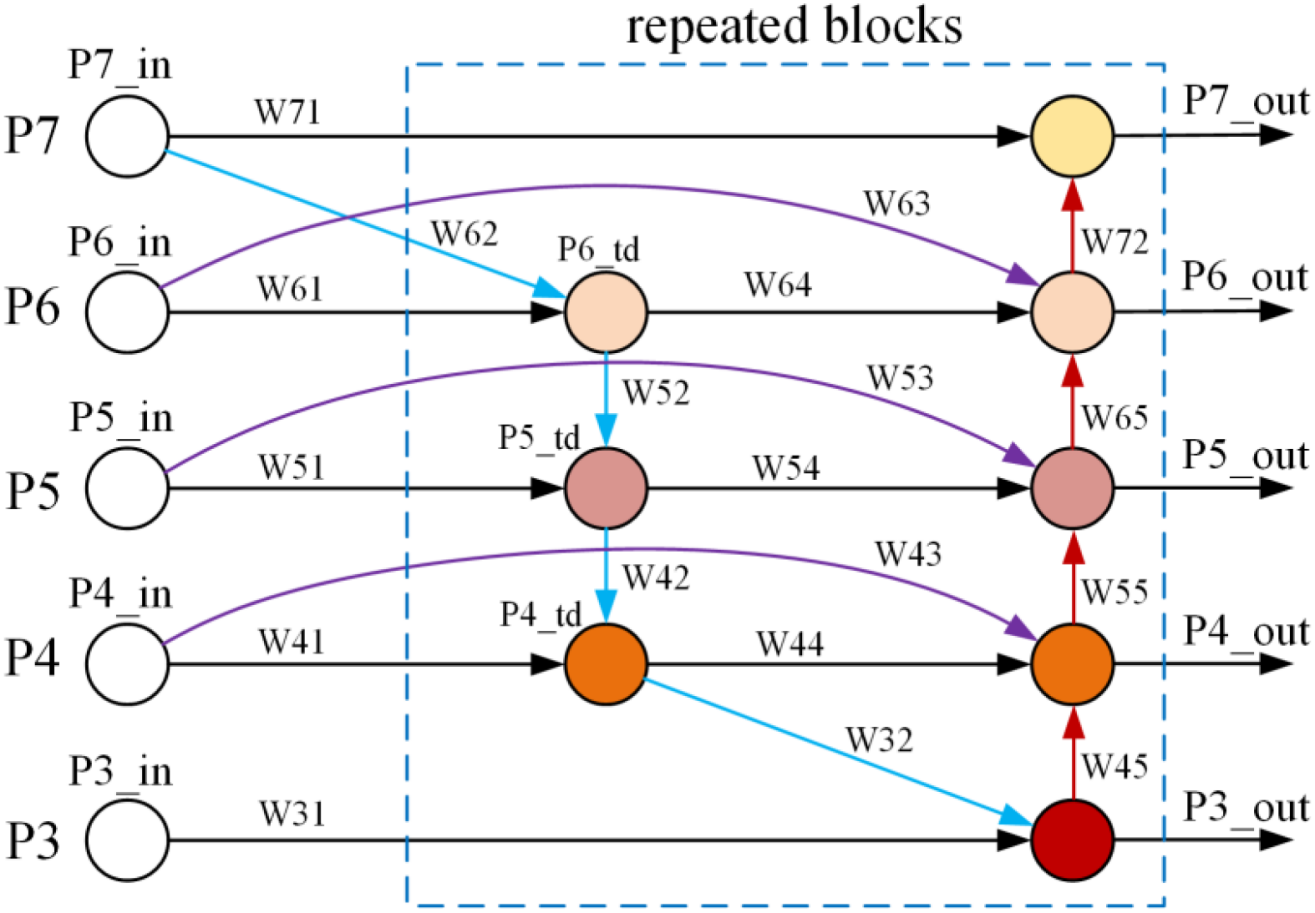
The architecture of BiFPN.

The architecture of the BiFPN module is illustrated in **Figure 6**. The symbols P3 through P7 represent the multi-scale output layers from the backbone network, where each layer produces a feature map with specific channel and spatial dimensions. For instance, the feature map from the P3 layer has a spatial size equal to the input image resolution divided by 2^3^, while P4 corresponds to the input resolution divided by 2^4^, and so forth, with P7 features being reduced by a factor of 2^7^. These features are denoted in the diagram as P3_in to P7_in. In the figure, uncolored circles represent feature maps, colored circles denote computational operators, and weighted connections indicate learnable weights W. The mathematical operation formula associated with each operator is shown in Equations 1–8.

(1) $$\begin{array}{c} P7_{out}=Conv\left\{\frac{P7_{in}*W71+Resize\left(P6_{out}\right)*W72}{W71+W72+\epsilon }\right\} \end{array}$$

(2) $$\begin{array}{c} P6_{td}=Conv\left\{\frac{P6_{in}*W61+Resize\left(P7_{in}\right)*W62}{W61+W62+\epsilon }\right\} \end{array}$$

(3) $$\begin{array}{c} P6_{out}=Conv\left\{\frac{P6_{in}*W63+P6_{td}*W64+Resize\left(P5_{out}\right)*W65}{W63+W64+W65+\epsilon }\right\} \end{array}$$

(4) $$\begin{array}{c} P5_{td}=Conv\left\{\frac{P5_{in}*W51+Resize\left(P6_{td}\right)*W52}{W51+W52+\epsilon }\right\} \end{array}$$

(5) $$\begin{array}{c} P5_{out}=Conv\left\{\frac{P5_{in}*W53+P5_{td}*W54+Resize\left(P4_{out}\right)*W55}{W53+W54+W55+\epsilon }\right\} \end{array}$$

(6) $$\begin{array}{c} P4_{td}=Conv\left\{\frac{P4_{in}*W41+Resize\left(P5_{td}\right)*W42}{W41+W42+\epsilon }\right\} \end{array}$$

(7) $$\begin{array}{c} P4_{out}=Conv\left\{\frac{P4_{in}*W43+P4_{td}*W44+Resize\left(P3_{out}\right)*W45}{W43+W44+W45+\epsilon }\right\} \end{array}$$

(8) $$\begin{array}{c} P3_{out}=Conv\left\{\frac{P3_{in}*W31+Resize\left(P4_{td}\right)*W32}{W31+W32+\epsilon }\right\} \end{array}$$

Within the overall architecture of BiFPN, dimension scaling operations align feature map resolutions through upsampling or downsampling, while convolutional layers (Conv) perform subsequent feature transformations. The introduction of the constant ϵ prevents denominator division by zero, thereby maintaining numerical stability and ensuring reliable training dynamics. Through this recursively efficient integration mechanism, BiFPN generates more discriminative and robust multi-scale feature information. This cross-scale aggregation capability significantly enhances the model’s detection accuracy, particularly when different scales of information are present in varying degrees of garlic damage. The BiFPN architecture better balances recognition performance for both large and small objects. Furthermore, the introduction of learnable weight mechanisms reduces the proposed model’s reliance on manual design and hyperparameter tuning, enhancing the network’s overall adaptability and learning capacity.

#### 2.2.5 SimAM attention mechanism

**Figure 7:**
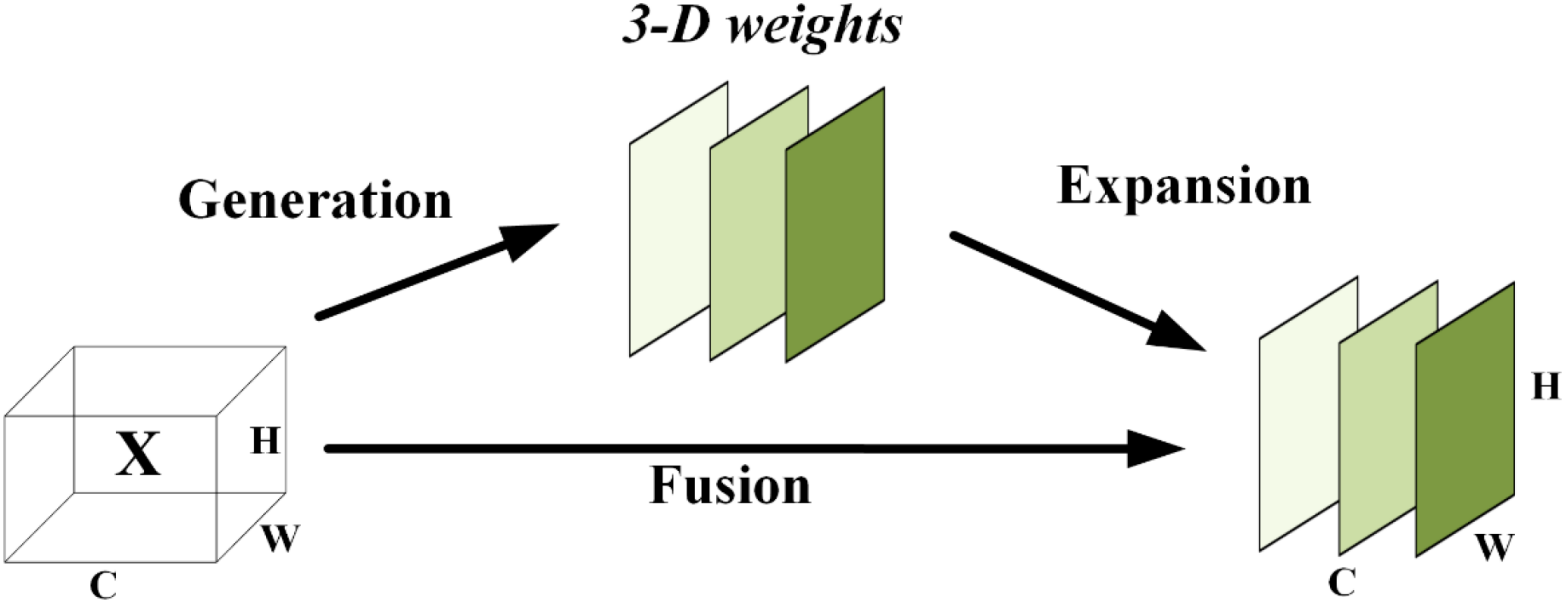
The architecture of SimAM.

SimAM (Simple Attention Module) (Yang et al., 2021) is a parameter-free attention mechanism proposed based on neuroscience-inspired saliency detection theory. Its core concept involves quantifying the importance of each neuron in feature maps through an energy function. SimAM treats individual neurons as independent processing units, estimating saliency by measuring the difference between a neuron and its surrounding environment. Neurons with lower energy are considered more information-rich. Based on this, SimAM automatically assigns three-dimensional attention weights to amplify relevant features and suppress redundant information—all without introducing any trainable parameters. Its structural diagram is shown in **Figure 7**.

The effectiveness of the SimAM module stems from its ability to apply three-dimensional attention weights, a mechanism that simultaneously integrates global features and local details. This enables the model to capture subtle variations and complex patterns within the data, thereby achieving more precise identification of garlic with varying degrees of damage. The module optimizes the attention mechanism through an energy function based on the principle of saliency, evaluating the uniqueness and relevance between neurons. Based on this concept, the energy function is formulated as follows:

(9) $$\begin{array}{c} e_{t}\left(w_{t},b_{t},y,x_{i}\right)=\;{\left(y_{t}-\hat{t}\right)}^{2}+\frac{1}{M-1}\sum_{i=1}^{M-1}{\left(y_{0}-{\hat{x}}_{i}\right)}^{2} \end{array}$$

Here, $\hat{t}$ = $w_{t}t+b_{t}$ and ${\hat{x}}_{i}$ = $w_{t}x_{i}+b_{t}$ represent linear transformations of *t* and $x_{i}$, respectively, where *t* and $x_{i}$ denote the target neuron and other neurons within a single channel of the input feature $X\in R^{B\times C\times H\times W}$. The index *i* spans the spatial dimensions, and *M = H×W* indicates the total number of neurons in that channel. The terms $w_{t}$ and $b_{t}$ correspond to the weight and bias of the transformation. All values in Equation 9 are scalars. Equation 9 reaches its minimum when $\hat{t}\;$ equals $\;y_{t}$ and all other ${\hat{x}}_{i}$ equal $y_{0}$, where $y_{t}$ and $y_{0}$ are two distinct values. By simplifying the expression, Equation 9 is equivalent to measuring the linear separability between the target neuron t and all other neurons in the same channel. For simplicity, binary labels (i.e., 1 and -1) are adopted, and a regularizer is incorporated into Equation 9. The resulting energy function is formulated as follows:

(10) $$\begin{array}{c} e_{t}\left(w_{t},b_{t},y,x_{i}\right)=\;\frac{1}{M-1}\sum_{i=1}^{M-1}{\left(-1-\left(w_{t}x_{t}+b_{t}\right)\right)}^{2}+{\left(1-\left(w_{t}t+b_{t}\right)\right)}^{2}+\lambda w_{t}^{2} \end{array}$$

Theoretically, each channel corresponds to *M* energy functions. Solving all these equations using iterative solvers such as SGD would be computationally expensive. However, Equation 10 admits a fast closed-form solution for $w_{t}$ and $b_{t}$, which can be derived as follows:

(11) $$\begin{array}{c} w_{t}=-\frac{2\left(t-\mu_{t}\right)}{{\left(t-\mu_{t}\right)}^{2}+2\sigma_{t}^{2}+2\lambda } \end{array}$$

(12) $$\begin{array}{c} b_{t}=-\frac{1}{2}\left(t+\mu_{t}\right)w_{t} \end{array}$$

In this context, $\mu_{t}=\frac{1}{M-1}\sum_{i=1}^{M-1}x_{i}$ and $\sigma_{t}^{2}=\frac{1}{M-1}\sum_{i}^{M-1}{\left(x_{i}-u_{t}\right)}^{2}$ represent the mean and variance computed across all neurons within the channel except the target neuron t. Since the closed-form solutions given in Equation 11 and Equation 12 are derived at the channel level, it is reasonable to assume that all pixels in a single channel follow the same distribution. Under this assumption, the mean and variance can be computed once across the entire set of neurons and reused for every neuron in the channel. This approach substantially reduces computational overhead by avoiding repeated iterative calculation of μ and σ for each spatial location. Consequently, the minimum energy can be efficiently computed as follows:

(13) $$\begin{array}{c} e_{t}^{*}=\frac{4\left({\hat{\sigma }}^{2}+\lambda \right)}{{\left(t-\hat{\mu }\right)}^{2}+2{\hat{\sigma }}^{2}+2\lambda } \end{array}$$

The mean and variance are estimated as $\hat{\mu }=\frac{1}{M}\sum_{i=1}^{M}x_{i}$ and ${\hat{\sigma }}^{2}=\frac{1}{M}\sum_{i=1}^{M}{\left(x_{i}-\hat{\mu }\right)}^{2}$, respectively. Equation 13 indicates that a lower energy value $e_{t}^{*}$ corresponds to greater discriminability of neuron *t* relative to its surrounding neurons, implying higher importance in visual processing. Thus, the significance of each neuron can be quantified as 1/ $e_{t}^{*}$. By incorporating a scaling operator for feature refinement, the overall optimization process of the module can be formulated as follows:

(14) $$\begin{array}{c} \tilde{X}=\;sigmoid\left(\frac{1}{E}\right)⊙X \end{array}$$

In Equation 14, *E* denotes the aggregation of all $e_{t}^{*}$ values across both channel and spatial dimensions, and ⊙ represents element-wise multiplication. A sigmoid function is applied to constrain excessively large values in *E*, and the result is multiplied by the original feature map *X* to produce a weighted feature map. This operation preserves the relative importance of each neuron, as the sigmoid function is a monotonic transformation.

## 3 Results and analysis

### 3.1 Experimental environment, parameter settings, and model evaluation metrics

All experiments were conducted on a workstation equipped with an Intel(R) Xeon(R) W-2245 CPU (3.9GHz) and an NVIDIA Quadro RTX 5000 GPU (16GB), running Windows 10. The software environment included Anaconda3 (2021.11), PyCharm as the compiler, and PyTorch 2.1.2 built on Python 3.8.19. The information on hyperparameters during the model training process is shown in **Table 1**. To ensure consistency, all algorithms were executed under identical hardware and software configurations.

**Table 1:** The setting of hyperparameters during the model training process.

Parameter	Value
Image size	640×640
Batch size	8
Learning rate	0.001
Epoch	300
Optimizer	Adam
Weight decay	0.0005
Momentum	0.937
Workers	16
Loss function	IoU

In this study, the performance of the model was evaluated based on several established metrics: recall (R), precision (P), F1 score (F1), average precision (AP), and mean average precision (mAP). Within the context of binary classification, the following standard definitions were applied: true positives (TP) correspond to positive instances correctly classified; false positives (FP) denote negative instances mistakenly identified as positive; false negatives (FN) refer to positive samples incorrectly classified as negative; and true negatives (TN) indicate negative samples accurately recognized. Precision is defined as the ratio of correctly predicted positive samples to all instances predicted as positive. Recall measures the proportion of actual positive samples that are correctly identified. F1-score is the harmonic mean of precision and recall, serving as a balanced evaluation metric that more comprehensively reflects the recognition performance of individual categories. mAP, on the other hand, calculates the average intersection-over-union score across all categories to holistically reflect overall detection performance. A higher score indicates greater accuracy across all categories. The specific calculation methods for these evaluation metrics are detailed in Equations 15–19.

(15) $$\begin{array}{c} P=\;\frac{TP}{TP+FP} \end{array}$$

(16) $$\begin{array}{c} R=\;\frac{TP}{TP+FN} \end{array}$$

(17) $$\begin{array}{c} F1=\;\frac{2\;\times Recall\;\times Precision}{Recall\;+Precision} \end{array}$$

(18) $$\begin{array}{c} AP=\;\int P\left(R\right)dR\;\times 100\% \end{array}$$

(19) $$\begin{array}{c} mAP=\;\frac{\sum_{i=1}^{N}AP_{i}}{N} \end{array}$$

When designing and evaluating lightweight deep learning models, we prioritize the following four lightweight evaluation metrics, which reflect the model’s computational efficiency, memory usage, and inference speed.

The number of parameters measures the total count of trainable variables, directly impacting memory consumption and computational load. Lightweight architectures typically employ efficient layer designs and parameter compression strategies to reduce storage and processing costs (Choi et al., 2025). Floating-point operations per second (FLOPs) estimate computational workload per forward pass. Effectively reducing FLOPs lowers energy consumption and enhances model applicability on low-power devices (Junaid et al., 2024). Model size refers to the storage space occupied by saved weights. Techniques like quantization and compression are commonly used to reduce model size, enabling deployment on embedded platforms (Yuan et al., 2013). FPS (frames per second) indicates the throughput of model inference, representing the number of images processed per second. Higher FPS supports real-time applications such as video analysis (Smistad et al., 2019). In this study, FPS is measured with a batch size of 1. In the results obtained from the ablation experiments and comparative experiments, all values represent the average of five independent assessments.

### 3.2 Ablation experiment results

As shown in **Table 2**, this ablation study systematically evaluated the effectiveness of the three modules introduced in this research. The baseline model YOLOv11n achieved mAP@50% of 64.94%, mAP@50-95% of 58.66%, and an F1 score of 59.35%, with 2.583 million parameters. When replacing the standard downsampling module with the ADown module, mAP@50% improved to 87.55%, while reducing parameters to 2.104 million and increasing inference speed from 144 fps to 156 fps. Using BiFPN alone slightly improved recognition accuracy while significantly reducing model parameters. The SimAM module substantially enhanced recognition performance without adding extra parameters, increasing accuracy by 11.00 percentage points to 59.86% and boosting the F1 score by 6.54 percentage points to 65.89%. Meanwhile, the combination of modules demonstrates significant synergistic effects. The collaborative application of ADown and BiFPN generates an ultra-low-parameter model achieving a high mAP@50%, highlighting their complementary roles in efficient feature extraction and fusion for garlic damage detection. Integrating ADown with SimAM further enhances performance, underscoring the powerful interaction between structured downsampling and parameter-free attention mechanisms. Finally, integrating all three enhancement strategies, Garlic-YOLO-DD achieves an impressive 92.58% mAP@50% while maintaining minimal parameters and high recognition speed. These results further validate the effectiveness of each module and the soundness of the overall architecture.

**Table 2:** Detailed procedure for the ablation experiment.

ADown	BiFPN	SimAM	mAP@50%	mAP@50-95%	P	R	F1	Parameters(M)	GFLOPs(G)	Weights (MB)	FPS (Frames/s)
			64.94	58.66	48.86	75.58	59.35	2.583	6.3	5.5	144
√			87.55	82.40	74.81	86.75	80.34	2.104	5.3	4.6	156
	√		66.91	61.23	50.46	79.64	61.78	1.923	6.3	4.2	151
		√	69.99	63.54	59.86	73.27	65.89	2.583	6.3	5.5	136
√	√		89.31	82.64	77.21	87.49	82.03	1.497	5.0	3.4	162
√		√	91.22	85.09	83.10	86.23	84.64	2.104	5.3	4.6	159
√	√	√	**92.58**	**86.59**	**87.48**	**84.26**	**85.84**	**1.497**	**5.0**	**3.4**	**167**

The bolded parts represent the optimal values.

**Figure 8:**
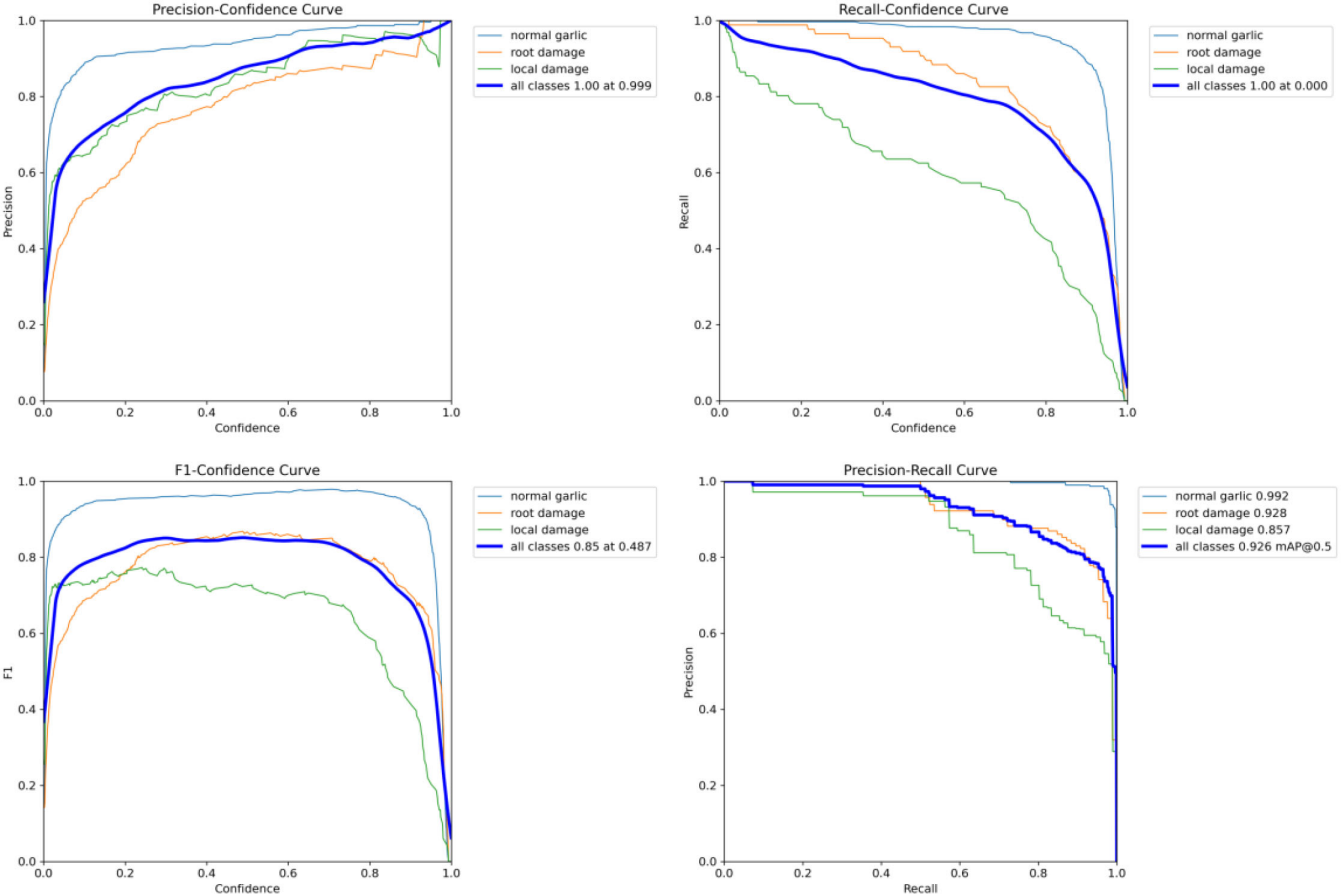
Precision-confidence curve, Recall-confidence curve, F1-confidence curve, and Precision-Recall curve of Garlic-YOLO-DD during training.

To systematically evaluate the performance of the Garlic-YOLO-DD model during training on normal garlic, partially damaged garlic, and root-damaged garlic, this study conducted a comprehensive comparison and analysis of its Precision-Confidence (P-C), Recall-Confidence (R-C), F1-Confidence (F1-C), and Precision-Recall (PR) curves. As shown in **Figure 8**, these curves reflect the model’s classification reliability, recall capability, and inter-class performance differences at various confidence thresholds. The P-C curves for all three categories maintained high values across all confidence levels, indicating the model’s stable high accuracy. The normal garlic curve rises steeply toward the upper-right quadrant, indicating strong alignment between predicted confidence and actual accuracy with minimal false positives. In contrast, the partially damaged and root-damaged curves exhibit slight fluctuations in the low-confidence range, reflecting greater morphological variability within these damage categories. However, as confidence increases, their precision consistently stabilizes at high levels, validating the model’s robust discrimination capability. The recall-confidence curve exhibits an expected monotonically decreasing trend as the confidence threshold increases. Root damage shows the gentlest initial decline, indicating its higher sensitivity. The F1-C curves all exhibit distinct peak states, aiding in determining the confidence interval for the optimal balance between precision and recall. The optimized threshold for normal garlic is the highest, while partially damaged garlic exhibits relatively lower thresholds. Ultimately, the PR curves further validate the model’s overall excellent performance. All curves cluster tightly in the upper-right quadrant, with the normal garlic category achieving the highest values and an ideal curve shape. Both damaged garlic categories also demonstrate relatively good recognition performance, confirming the model’s effectiveness in damage identification. Notably, the Garlic-YOLO-DD model achieved the best recognition performance on normal garlic and demonstrated high model performance on the other two damage states as well.

**Figure 9:**
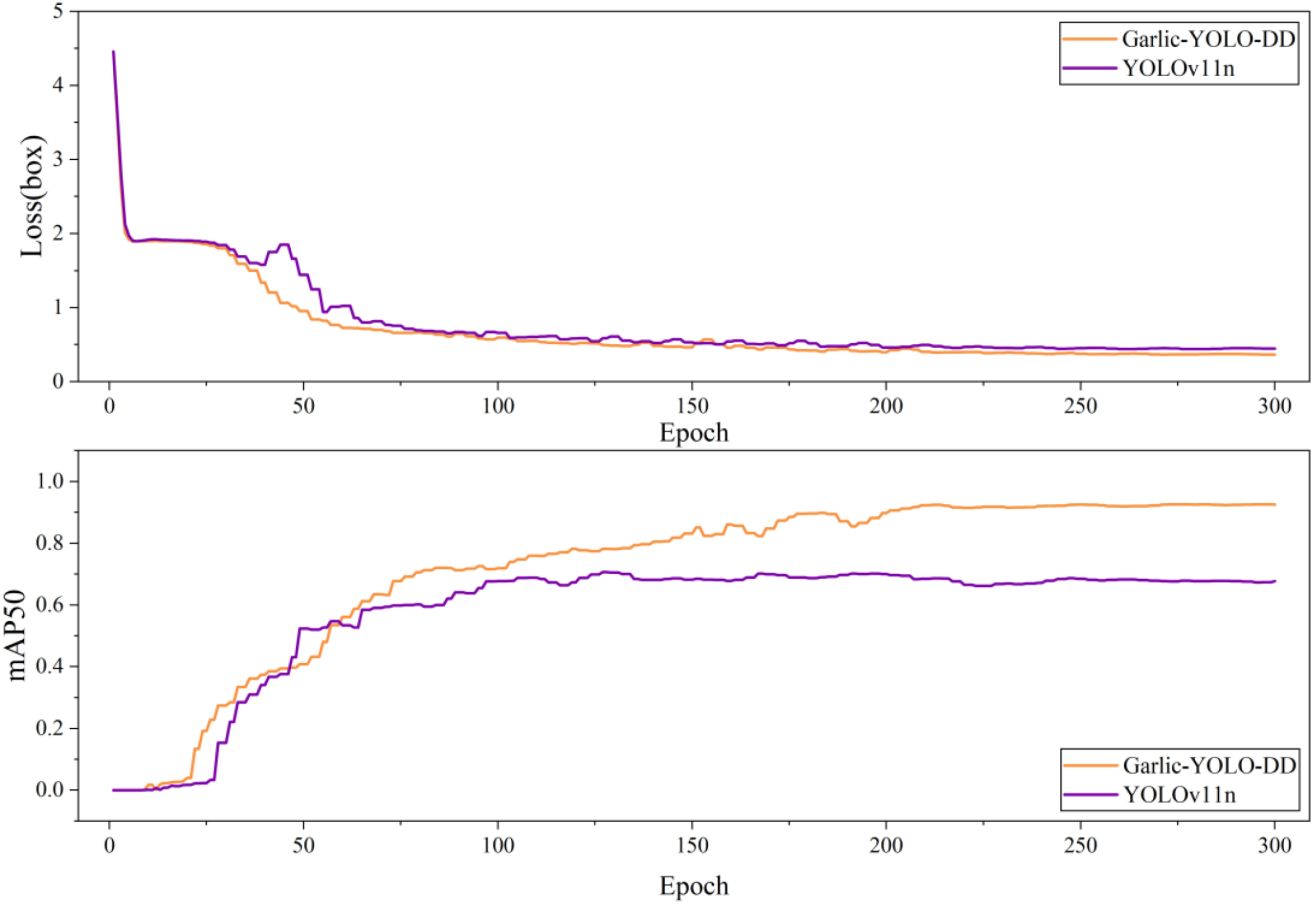
Comparison of the bounding box loss curves before and after model improvement, and comparison of mAP@50% before and after model improvement.

**Figure 9** presents a comparative analysis between the proposed Garlic-YOLO-DD model and the baseline YOLOv11n on the training cycle validation set, focusing on the bounding box regression loss curve and mAP50%. The results clearly demonstrate that Garlic-YOLO-DD exhibits superior convergence stability and achieves a lower final loss value. The baseline YOLOv11n model exhibits high loss values during early training, accompanied by persistent fluctuations throughout the training process, ultimately converging to a relatively high loss value. This pattern indicates potential challenges in optimizing localization for this task. In contrast, Garlic-YOLO-DD’s loss curve demonstrates clear advantages: faster early descent rates, smoother and more stable convergence without significant fluctuations, and a lower final loss value. The substantial reduction in regression loss indicates that Garlic-YOLO-DD’s architectural optimizations effectively enhance the model’s localization capabilities, enabling more precise bounding box coordinates for garlic recognition. In terms of mAP@50% curve comparisons, the Garlic-YOLO-DD model demonstrates significantly superior convergence characteristics and final performance compared to YOLOv11n. Specifically, YOLOv11n exhibits a relatively flat initial ascent phase in its convergence curve, maintaining a low level throughout the entire training cycle, resulting in a lower final stable value for mAP@50%. In contrast, the Garlic-YOLO-DD curve exhibits a steeper initial ascent phase, indicating faster feature learning rates and gradient propagation efficiency achieved through structural optimization. More importantly, this curve not only converges faster initially but also maintains higher performance throughout the entire training process. This comparative result validates the effectiveness of the model’s structural design from a training dynamics perspective.

**Figure 10:**
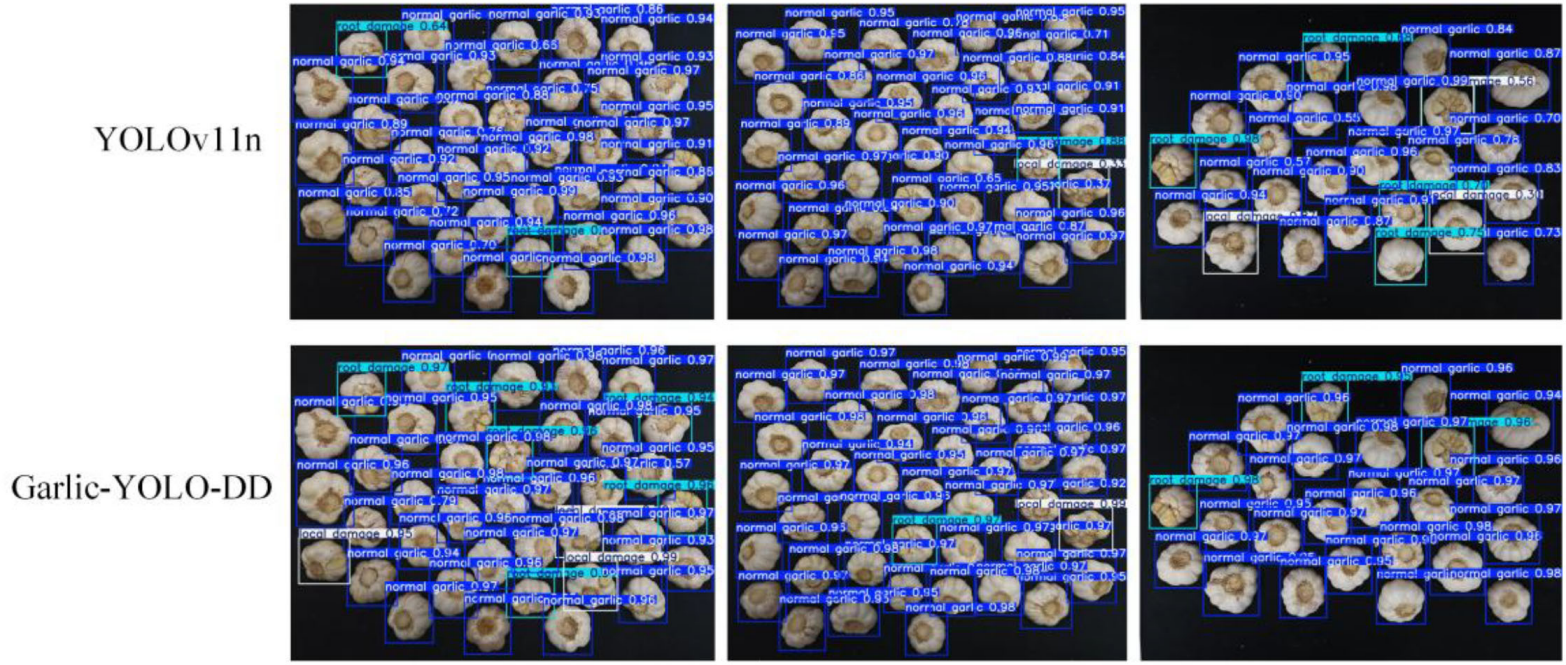
Comparison of recognition performance before and after model improvement.

To visually assess the performance gap between the baseline model YOLOv11n and Garlic-YOLO-DD, **Figure 10** presents a comparison visualization of detection results on the test set before and after model refinement. Most notably, YOLOv11n generates a high number of false detections, misclassifying damaged garlic as intact garlic, reflecting its insufficient feature discrimination capability. In contrast, Garlic-YOLO-DD demonstrates outstanding detection performance on the test set. This model significantly reduces false detection rates and successfully identifies damaged garlic instances. This improvement can be attributed to the SimAM module, which enhances feature attention, while the BiFPN architecture improves multi-scale feature integration. In summary, this visual analysis strongly supports the findings of the quantitative study, confirming that Garlic-YOLO-DD not only outperforms the baseline model numerically but also demonstrates higher reliability in the testing task.

### 3.3 Comparative experiment results

To comprehensively evaluate the performance of the proposed model, this study conducted comparative experiments with nine classic object detection algorithms, including Faster R-CNN, RT-DETR, YOLOv5n, YOLOv6n, YOLOv8n, YOLOv10n/s, YOLOv11n, and YOLOv12n. As shown in **Table 3**, all algorithms were evaluated under identical experimental conditions. The proposed Garlic-YOLO-DD model demonstrated significant advantages in detection accuracy, achieving the highest recognition performance with an mAP@50 value of 92.58%, surpassing the second-best YOLOv10s (89.06%) by 3.52 percentage points. Furthermore, Garlic-YOLO-DD achieved an accuracy of 87.48%, a recall rate of 84.26%, and an F1 score of 85.84%, also delivering excellent recognition performance. It is worth emphasizing that Garlic-YOLO-DD achieves significant accuracy improvements while maintaining highly lightweight characteristics. The model contains only 1.497 million parameters, representing a 31.4% reduction compared to the already impressive YOLOv5n. Garlic-YOLO-DD operates at a computational cost of 5.0 GFLOPs, lower than all comparison models. Its model weight file is only 3.4MB, representing just 20.5% of the YOLOv10s model. In terms of inference efficiency, this model achieves 167 frames per second, surpassing all lightweight counterparts in the study and demonstrating a significant advantage in throughput.

**Table 3:** Comparative experiments with classic models.

Models	mAP@50%	mAP@50-95%	P	R	F1	Parameters(M)	GFLOPs(G)	Weights (MB)	FPS (Frames/s)
Faster R-CNN	60.63	52.12	51.17	68.46	59.76	56.491	222.0	216.5	62
RT-DETR(ResNet18)	67.16	54.43	62.15	68.24	65.37	19.912	56.9	38.6	96
YOLOv5n	56.59	50.11	46.11	70.19	55.66	2.182	5.8	4.7	147
YOLOv6n	87.44	83.03	76.96	85.75	81.12	4.155	11.5	8.6	118
YOLOv8n	61.71	55.32	55.82	63.81	59.55	2.685	6.8	5.6	141
YOLOv10n	63.08	58.08	55.05	65.90	59.99	2.266	6.5	5.8	155
YOLOv10s	89.06	85.38	79.44	81.52	80.47	7.219	21.4	16.6	102
YOLOv11n	64.94	58.66	48.86	75.58	59.35	2.583	6.3	5.5	144
YOLOv12n	60.12	54.88	54.22	62.11	57.90	2.557	6.3	5.5	145
Garlic-YOLO-DD	**92.58**	**86.59**	**87.48**	**84.26**	**85.84**	**1.497**	**5.0**	**3.4**	**167**

The bolded parts represent the optimal values.

To investigate the impact of input image size on model detection performance, this study systematically evaluated Garlic-YOLO-DD’s performance in identifying garlic damage effects at different resolutions. As shown in **Table 4**, when the resolution increased from 240×240 pixels to 960×960 pixels, a distinct nonlinear relationship emerged between input scale and model performance. The optimal input size was determined to be 640×640 pixels, where the model achieved the best comprehensive metrics: mAP@50% of 92.58%, mAP@50-95% of 86.59%, precision of 87.48%, recall of 84.26%, and an F1 score of 85.84%. At this resolution, the model demonstrated the highest localization accuracy and classification performance.

**Table 4:** The impact of different image sizes on model results.

Image size	mAP@50%	mAP@50-95%	P	R	F1
240×240	84.33	77.96	76.48	76.24	76.36
340×340	90.22	83.77	83.95	85.49	84.71
460×460	82.65	77.80	73.10	81.57	77.10
520×520	84.37	77.88	80.25	76.12	78.13
640×640	**92.58**	**86.59**	**87.48**	**84.26**	**85.84**
760×760	89.43	82.07	80.26	88.52	84.19
820×820	89.59	83.37	83.50	79.50	81.45
960×960	88.24	81.18	83.57	81.36	82.45

The bolded parts represent the optimal values.

It is noteworthy that the model’s recognition performance did not improve with increased resolution. For instance, increasing the input size from 340×340 to 460×460 resulted in a noticeable decline in recognition performance, indicating that the model exhibits sensitivity to different scales across resolutions. At 760×760 pixels, recall improved to 88.52%, but precision dropped to 80.26%, suggesting a rising false alarm rate. These results further validate the importance of balancing feature detail when selecting input image dimensions. The 640×640 resolution provides sufficient spatial information for reliable garlic damage detection while avoiding performance degradation caused by excessive computation, offering practical guidance for model deployment in real-world scenarios.

**Figure 11:**
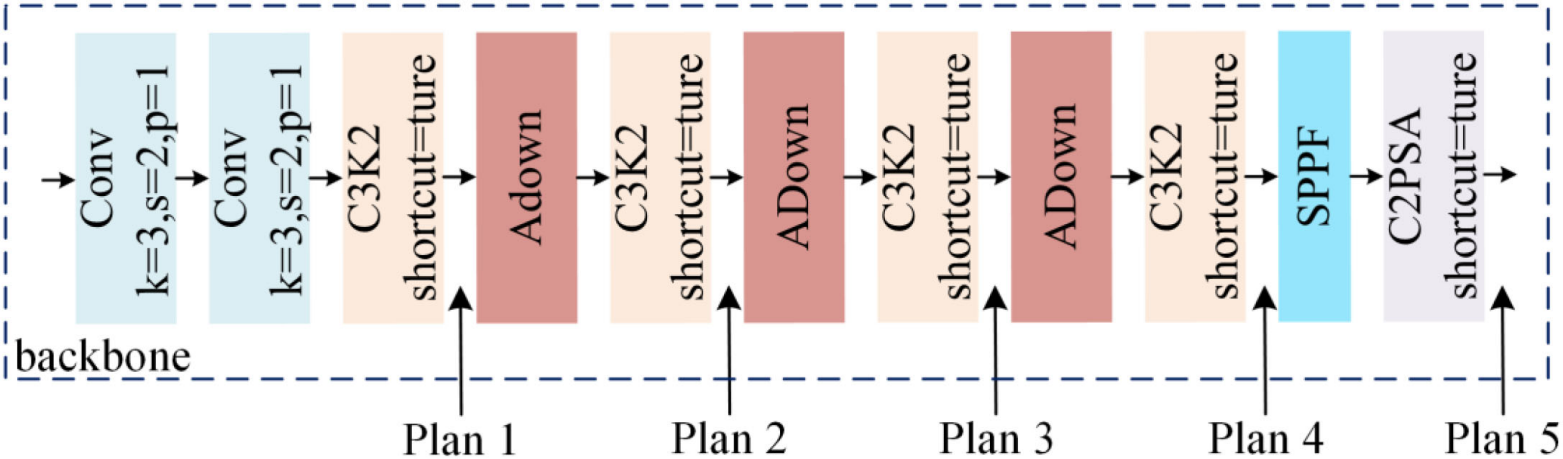
Schematic diagram showing the attention mechanism positioned at different locations.

As shown in **Figure 11**, this study investigates the impact of embedding the attention mechanism at different depths within the backbone network on model performance. Five schemes were designed for comparison, ranging from shallow to deep layers of the model backbone. Experimental results demonstrate that the embedding position of the attention mechanism significantly influences Garlic-YOLO-DD’s performance in garlic damage detection, with performance improvements exhibiting a clear positive correlation with embedding depth.

**Table 5** shows the performance of the Garlic-YOLO-DD model when the SimAM attention mechanism is embedded at different depths of the main network. When SimAM is only placed at the shallow stage (Plan 1), the model achieves mAP@50% of 82.36% and an F1 score of 77.07%, indicating a limited improvement. As the module is embedded at deeper levels, the performance gradually improves. Plan 2 and Plan 3 increase mAP@50% to 84.22% and 86.72% respectively, corresponding to F1 values of 78.15% and 81.28%. This indicates that applying the attention mechanism to deeper features can capture more semantic distinction information. When SimAM is embedded at a deeper level (Plan 4), mAP@50% reaches 91.95%, and the F1 score rises to 85.30%, which is 9.59 and 8.23 percentage points higher than Plan 1, respectively. When SimAM is placed at the bottom layer (Plan 5), Garlic-YOLO-DD achieves the best results for garlic damage recognition, with mAP@50% reaching 92.58%, mAP@50-95% reaching 86.59%, precision rate of 87.48%, recall rate of 84.26%, and F1 score of 85.84%. These results indicate that embedding the attention mechanism at a deeper level enables the model to more effectively focus on the key areas for damage detection.

**Table 5:** Comparison of results from placing attention mechanisms in different positions.

Plan	mAP@50%	mAP@50-95%	P	R	F1
Plan 1	82.36	76.81	73.15	81.43	77.07
Plan 2	84.22	77.52	81.36	75.18	78.15
Plan 3	86.72	81.29	76.93	86.15	81.28
Plan 4	91.95	86.23	83.65	87.02	85.30
Plan 5(my)	**92.58**	**86.59**	**87.48**	**84.26**	**85.84**

The bolded parts represent the optimal values.

## 4 Discussion

The Garlic-YOLO-DD model proposed in this study demonstrates outstanding performance in garlic damage detection tasks. It exhibits exceptional comprehensive advantages in accuracy, lightweight design, and inference speed, providing an innovative solution for lightweight visual detection of garlic damage. The following sections will discuss the model’s performance, structural design, application potential, and limitations.

Firstly, Garlic-YOLO-DD maintains extremely low parameters and computational costs while achieving mAP@50% of 92.58% and F1 score of 85.84%, significantly outperforming several mainstream lightweight models. This result further validates the effective combination of the introduced modules. Notably, with a 42.04% reduction in parameters, the mAP@50% of this model has increased by 27.64% compared to YOLOv11n, fully demonstrating that the architecture design has significantly improved the efficiency of parameter utilization.

Secondly, ablation studies demonstrate the critical role of the introduced modules in this research. The ADown module, through its asymmetric convolution design, reduces computational cost while preserving feature information during downsampling, significantly boosting model accuracy while substantially lowering computational overhead. The parameter-free SimAM attention mechanism enhances the model’s ability to focus on damaged areas of garlic without substantially increasing computational burden. BiFPN enhances multi-scale representations through weighted bidirectional feature fusion, improving consistency and differentiation across varying damage sizes. Validation experiments with five attention placement schemes reveal that embedding the attention mechanism deeper within the model backbone captures more damage-related information, leading to more substantial performance gains. Comparative experiments on the image resolution input to the model indicate that it performs optimally at 640×640 pixels. This suggests that at this resolution, the model can capture more damage information in garlic bulbs. Lower or higher resolutions reduce the model’s effectiveness in identifying damaged areas in garlic bulbs.

Although the base model YOLOv11n used in this study did not achieve optimal performance in the garlic recognition task, selecting YOLOv11n as the base model more clearly highlights the effectiveness of subsequent enhancement measures. It is worth noting, however, that the recognition performance of the proposed improvement strategy may vary across different base models. This phenomenon warrants further investigation in future research. Comparative experiments confirm that Garlic-YOLO-DD not only surpasses stronger base models like YOLOv6n in accuracy but also maintains a significant advantage in terms of model complexity. Despite the promising results of this study, several limitations remain that will be addressed in future research. First, the training and validation data were collected under specific imaging conditions, introducing singularity and uncertainty. Future work should validate the model’s ability to identify garlic damage severity under varying lighting conditions, garlic varieties, and camera hardware. Additionally, the current model identifies only three distinct damage types. Expanding its capability to recognize more damage categories and anomalies—such as moldy, sprouted, or diseased garlic—would enhance its value. Second, while the Garlic-YOLO-DD model demonstrates excellent time complexity, space complexity, and computational efficiency, its real-time performance on resource-constrained edge devices requires further validation. Furthermore, a real-time garlic conveying and sorting device will be incorporated into future research. As garlic bulbs move along a conveyor belt, the system captures and analyzes multi-angle images in real time, enabling millisecond-level precise identification of root or skin damage. Upon detecting defects, the sorting device automatically rejects non-compliant bulbs. This solution replaces manual inspection, enhances sorting consistency and throughput, reduces labor costs, and eliminates direct contact with agricultural products. Consequently, the model provides a reliable technical pathway for intelligent post-harvest processing of garlic, demonstrating how computer vision technology can translate into tangible agricultural productivity.

## 5 Conclusion

The lightweight garlic damage assessment model Garlic-YOLO-DD proposed in this study integrates the ADown downsampling module, SimAM parameter-free attention mechanism, and BiFPN feature fusion architecture. This approach significantly reduces parameter count and computational complexity while substantially improving recognition accuracy. Experimental results demonstrate that the model achieves an outstanding mAP50% of 92.58% on a self-built garlic damage dataset, requiring only 1.497 million parameters and delivering an inference speed of 167 frames per second. Its overall performance exhibits a clear advantage over current mainstream lightweight detection models. Garlic-YOLO-DD provides a computer vision solution for rapid, non-destructive detection of garlic damage, demonstrating promising practical application prospects.

## Data Availability

The original contributions presented in the study are included in the article/supplementary material. Further inquiries can be directed to the corresponding author.
